# WTAP mediates the anti-inflammatory effect of *Astragalus mongholicus polysaccharide* on THP-1 macrophages

**DOI:** 10.3389/fphar.2022.1023878

**Published:** 2022-10-07

**Authors:** Haijiao Long, Haiyue Lin, Pan Zheng, Lianjie Hou, Ming Zhang, Shuyun Lin, Kai Yin, Guojun Zhao

**Affiliations:** ^1^ Xiangya Hospital, Central South University, Changsha, China; ^2^ The Sixth Affiliated Hospital, Guangzhou Medical University, Qingyuan, China; ^3^ College of Pharmacy, Guilin Medical University, Guilin, China; ^4^ Department of Cardiology, The Second Afliated Hospital of Guilin Medical University, Guangxi Key Laboratory of Diabetic Systems Medicine, Guilin, China

**Keywords:** APS, M6A, WTAP, macrophage inflammation, IL-6

## Abstract

**Background:** *Astragalus mongholicus polysaccharides* (APS) have anti-inflammatory, antioxidant and immunomodulatory effects. Recent studies have demonstrated the epigenetic regulation of N6-methyladenosine (m^6^A) in the development of inflammation. However, the effect of APS on m^6^A modification is unclear. Here, for the first time, we investigate the mechanism of m^6^A modification in APS regulation of THP-1 macrophage inflammation.

**Methods:** We treated LPS-induced THP-1 macrophages with APS at different concentrations and times, and detected IL-6 mRNA and protein levels by quantitative real-time PCR (qRT-PCR) and western blot, respectively. The m^6^A modification level was detected by m^6^A quantification kit. The proteins that regulate m^6^A modification were screened by western blot. Wilms’ tumor 1-associating protein (WTAP) was overexpressed in APS-treated THP-1 macrophages and the m^6^A modification level and IL-6 expressions were detected.

**Results:** These findings confirmed that APS significantly abolished LPS-induced IL-6 levels in THP-1 macrophages. Meanwhile, APS reduced m^6^A modification levels and WTAP gene expression in THP-1 macrophages. Further overexpression of WTAP can significantly reverse APS-induced m^6^A modification level and IL-6 expression. Mechanistically, APS regulates IL-6 expression through WTAP-mediated p65 nuclear translocation.

**Conclusion:** Overall, our study suggested that WTAP mediates the anti-inflammatory effect of APS by regulating m^6^A modification levels in THP-1 macrophages. This study reveals a new dimension of APS regulation of inflammation at the epigenetic level.

## Introduction

N6-Methyladenine (m^6^A) modification is an epigenetic modification and is the most common and abundant modification of RNA molecules in eukaryotes (Marnef and Legube, 2020). In mammalian cells, m^6^A methylation was shown to be enriched in mRNAs containing 3–5 m^6^ A modifications per mRNA (Wang et al., 2014). RNA m^6^A modification has been shown to play important roles in regulating RNA splicing, translation, stability, translocation and high-level structure (Jiang et al., 2021). Studies have shown that m^6^A methylation of RNA is mediated by a methyltransferase complex consisting of methyltransferase 3 and 14 (METTL3, METTL14) and wilms’ tumor 1-associating protein (WTAP). M^6^A methylation is reversed by two demethylases: fat mass and obesity-associated (FTO) and alkylation repair homolog 5 (ALKBH5) (Xu et al., 2020). Recently, research has shown that m^6^A modifications occur in most species and play important roles in the development of many diseases, including tumors (Liu et al., 2020), neurological (Li et al., 2019) and cardiovascular diseases (Paramasivam et al., 2020), etc.

Monocytes and macrophages are major players in many chronic inflammatory diseases (Aguzzi et al., 2020). Macrophages initiate and maintain inflammation, produce inflammatory cytokines (TNF-α, IL-1β and IL-6) (Tabas and Bornfeldt, 2016). At present, multiple studies have reported that defects in m^6^A modification affect the development of inflammation in mouse and human macrophages (Li et al., 2022). However, no targeted therapy has been shown to ameliorate macrophage inflammatory responses through m^6^A modifications.

*Astragalus mongholicus polysaccharide* (APS) is a water-soluble heteropolysaccharide obtained by extracting, concentrating and purifying the dried roots of leguminous plants Astragalus mongolica or Astragalus membranaceus. The molecular formula of APS is C_10_H_7_CIN_2_O_2_S. APS is one of the most well-known herbal medicines that is widely used in China (Yuan et al., 2009). In addition to its antioxidant (Jia et al., 2019), antitumor (Liao et al., 2020) and immunomodulatory (Shao et al., 2006) effects, several studies have shown that APS can be used to treat chronic inflammatory diseases, including atherosclerosis (Chen et al., 2015). However, whether APS can exert anti-inflammatory effects by regulating the m^6^A modification remains unclear.

In this study, we investigated the effect of APS on RNA m^6^A modification during LPS-induced inflammation in THP-1 macrophages. It was further shown that APS affected the m^6^A modification level through the m^6^A “writer” protein WTAP. Overall, our data suggest that WTAP mediates the anti-inflammatory effects of APS by regulating m^6^A modification levels in THP-1 macrophages. This study reveals a new mechanism by which APS regulates inflammation at the epigenetic level.

## Materials and methods

### Cell culture and APS administration

THP-1 was purchased from ATCC (TIB-202) and maintained in RPMI 1640 medium in 10% FBS with penicillin and streptomycin at 37 °C in humidified 5% CO_2_. The macrophage-like state was obtained by treating the THP-1 monocytes for 48 h with 100 nM PMA (MCE, United States). The culture medium was removed, and then the cells were exposed to various concentrations of APS (50–500 μg/ml, Solarbio, CHN) diluted in culture medium for different time intervals at 37°C. Subsequently, the cells were stimulated with 10 μg/ml lipopolysaccharide (LPS, Sigma, United States) for 4 h until further analysis.

### Cell viability assay

Cell toxicity indicated as the percentage of cell viability was determined using the standard MTT assay (Sigma-Aldrich, Germany). THP-1 macrophages were seeded in 96-well (1 × 10^5^ cells/well) plates for 48 h and then treated with APS (0–500 μg/ml) for 24 h. After treatment, 20 μL MTT solution was added to each well and incubated for 4 h. Next, 150 μL of dimethyl sulfoxide was added to each well, and then the absorbance of each well was measured at 490 nm using a microplate reader. Cell viability was normalized to the control group.

### RNA preparation and quantitative real-time polymerase chain reaction

Total RNA was extracted from cultured cells with TRizol reagent (TAKARA BIO INC., Osaka, Japan), followed by reverse transcription into cDNA with the PrimeScript RT reagent Kit with gDNA Eraser (TAKARA BIO INC., Osaka, Japan). qRT-PCR was performed with a SYBR Green Premix Pro Taq HS qPCR Kit (ACCURATE BIOLOGY, Hunan, China). Relative changes in mRNA levels were determined by the ΔΔCT method, using GAPDH levels as controls. Primer pair sequences are shown in Supplementary Table S1.

### Preparation of whole cell lysates fractions

Cells were washed in cold PBS and harvested as following. Whole cell lysates were prepared by lysing the cells for 30 min on ice with a modified RIPA lysis buffer (Beyotime, Shanghai, China) containing 1:100 phenylmethanesulfonyl fluoride (PMSF, Beyotime, Shanghai, China). Cytosolic and nuclear fractions were obtained using the Nuclear/Cytosol Fractionation Kit (Biovision, United States), according to the manufacturer’s instructions. Protein amounts were measured by a protein assay kit (BCA; Beyotime, Shanghai, China).

### Western blotting

Cellular proteins were resolved by SDS-PAGE and transferred onto nitrocellulose membranes (Merck Millipore Ltd., Melbourne, Australia). After being blocked by 5% non-fat milk in TBST for 1 h, membranes were incubated with corresponding primary antibodies at 4°C overnight. Washed by TBST for three times, they would be incubated with HRP-conjugated secondary antibodies for 1 h at room temperature. The immunoblots were detected with an imaging system (Bio-Rad, United States) using enhanced Immobilon Wrstern Chemiluminescent HRP Substran (Millipore, Boston, United States). GAPDH were selected to be the loading controls. Primary antibodies are shown in Supplementary Table S2.

### Small interfering RNA (siRNA) transfection

Briefly, THP‐1 cells were seeded in 6-well plates, and 48 h later, WTAP siRNA (150 pM) was introduced to the cells using GP-transfect-Mate (Genepharma, Shanghai, China) according to the manufacturer’s instructions. Non‐silencing siRNA and siRNAs specific for WTAP were transfected. Cells were harvested after 24 h for qRT-PCR examination or after 48 h for western blotting. The selected sequences for knockdown as follow: si-WTAP sense were 5′-GCU​UGG​ACU​CAA​GUG​UAA​ATT-3′, si-WTAP antisense were5′-UUUACACUUGAGUCCAAGCTT-3′.

### Plasmid transfection

All new plasmids were generated using standard molecular biology methods and were verified by sequencing. Overexpressing plasmid (1.2 μg) (WeiZhen Biosciences, Shangdong, China) of indicated genes were transfected into THP-1 macrophages using Attractene Transfection Reagent (Qiagen, Germany) according to the manufacturer’s instructions. For each well (6-well plates), plasmid DNA was mixed with Attractene Transfection Reagent (with 1.2 μg–4.5 μL ratio) in 100 μL serum-free medium. After incubating the mixture at room temperature for 15 min, 1.9 ml per well of fully supplemented RPMI 1640 medium was added. 100 μL of the resulting mixture was added to the wells that had 1.9 ml of cell medium. The plate was gently shaken to ensure that the reagents were well mixed. The efficiency of WTAP plasmid transfection using Attractene Transfection Reagent was as high as about 80%.

### Quantification of the m^6^A modification

Total RNA was isolated using TRIzol reagent (TAKARA BIO INC., Osaka, Japan) according to the manufacturer’s instructions. The RNA quality was analyzed using the NanoDrop2000 system. The change in global m^6^A levels in the mRNA was measured using the EpiQuik™ m^6^A RNA Methylation Quantification Kit (Colorimetric; Epigentek, P-9005–48) following the manufacturer’s protocol. For total RNA, 200 ng of RNA per sample was transferred to corresponding wells in a 96-well plate. All samples were performed in triplicate. Both negative and positive controls as well as a standard curve in the range of 0.02 ng–1 ng of m^6^A were included as recommended by the manufacturer. The capture antibody solution and detection antibody solution were then added to the assay wells separately using a suitable diluted concentration. The m^6^A levels were colorimetrically quantified by reading the absorbance at a wavelength of 450 nm, and then calculations were performed based on the standard curve.

### mRNA stability

To evaluate mRNA stability, THP-1 macrophages were treated with 4 μM Actinomycin D (Act D, MedChemExpress, United States) during indicated times and harvested. Then mRNA expression of IL-6 was determined by qRT-RCR analysis.

### Protein stability

To evaluate protein stability, THP-1 macrophages were treated with 10 μM cycloheximide (CHX, MedChemExpress, United States) during indicated times and harvested. Then protein expression of IL-6 was determined by western blot analysis.

### Immunofluorescence

THP-1 macrophages were cultured at 37°C, and the cells were washed with PBS and fixed in 500 μL 4% paraformaldehyde for 20 min at room temperature (RT). The cells were washed three times with PBS for 5 min each, permeabilized in 0.1% Triton X-100 in PBS for 30 min at RT. The cells were washed three times with PBS for 5 min each, and blocked with diluted goat serum (1:10) for 1 h with gentle shaking at RT. The cells were then incubated in blocking buffer containing diluted primary antibodies (anti - P65) overnight at 4°C and washed three times with PBS for 5 min each time. The cells were incubated with secondary antibodies in blocking buffer for 1 h at 37°C in the dark, and then washed three times with PBS for 5 min each time. The cells were incubated with DAPI (0.1 μg/ml) for 10 min, washed three times with PBS for 5 min, and fixed. Images were acquired using a confocal microscope (Zeiss, German).

### Statistical analysis

The results are presented as means ± SEM. Statistical analyses were performed using the GraphPad Prism version 8.0.1. Two-group comparisons were performed by unpaired two-tailed Student’s t test. *p*-values <0.05 were considered statistically significant. All experiments were performed at least three times.

## Results

### APS abrogated LPS-induced IL-6 gene expression in THP-1 macrophages

**FIGURE 1:**
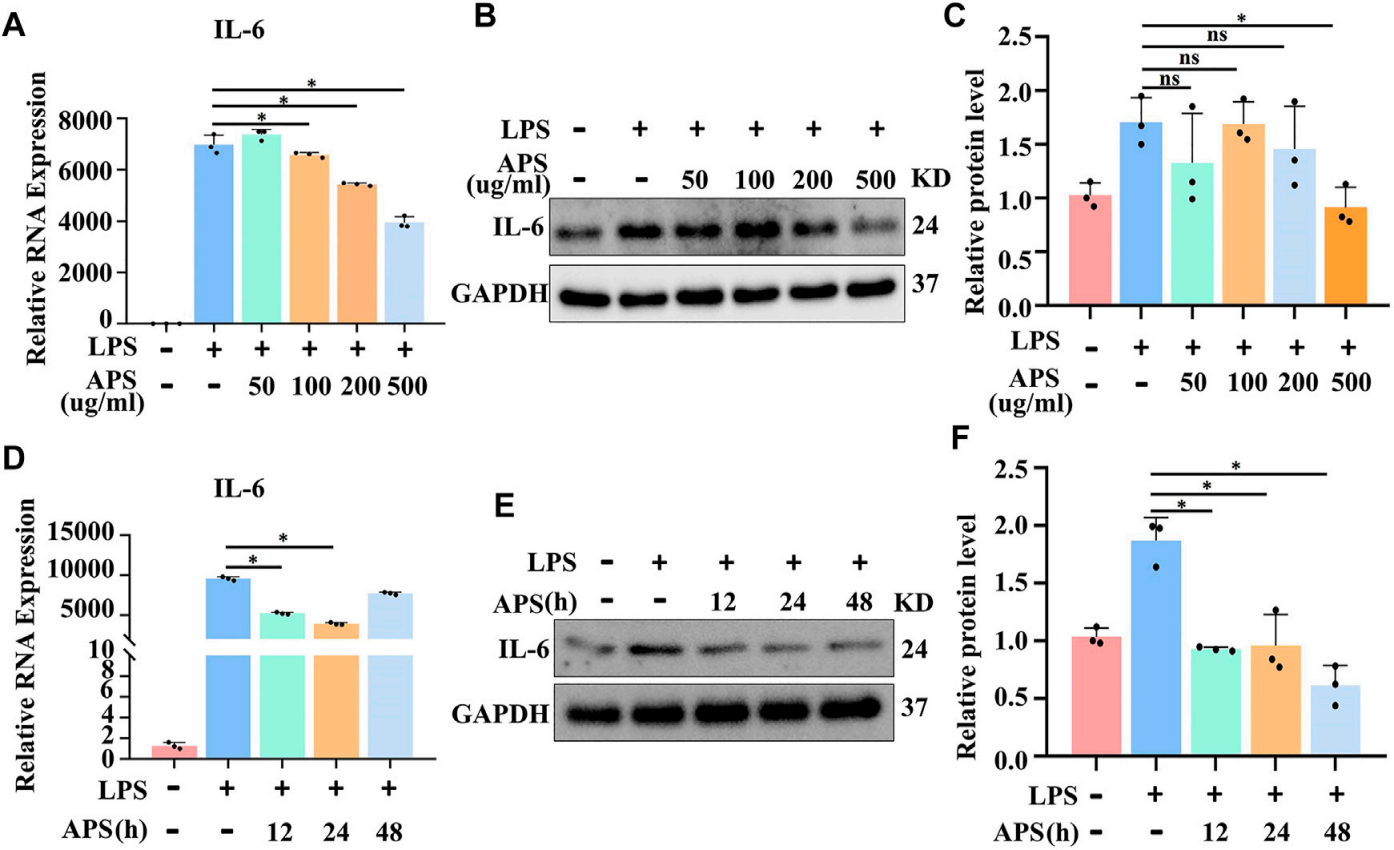
APS abrogated LPS-induced IL-6 gene expression in THP-1 macrophages. THP-1 macrophages were treated with APS (50, 100, 200, 500 μg/ml) for 24 h and then induced with LPS (10μg/ml) for 4 h **(A)** IL-6 mRNA was detected by qRT-PCR. **(B)** IL-6 protein was detected by western blot. **(C)** Densitometry quantification of protein. THP-1 macrophages were treated with APS (500 μg/ml) for different time (0, 12, 24, 48 h) and then induced with LPS (10 μg/ml) for 4 h **(D)** IL-6 mRNA was detected by qRT-PCR. **(E)** IL-6 protein was detected by western blot. **(F)** Densitometry quantification of protein. ^*^ *p* < 0.05 compared with LPS group. Data represent the mean ± SEM. n = 3 per group.

It has since been clarified that IL-6 is a critical factor in inflammation. To assess the effect of APS on IL-6 gene expression in THP-1 macrophages, we pretreated THP-1 macrophages with different concentrations (50, 100, 200 and 500 μg/ml) of APS for 24 h and stimulated them with LPS (10 μg/ml) for 4 h. First, we performed the MTT assay and found that 50–500 μg/ml APS did not show any cytotoxicity to THP-1 cells over 24 h (Supplementary Figure S1), which allowed us to rule out nonspecific cytotoxicity as a possible explanation for the decrease in cytokine output. Second, relevant inflammatory factor expression was measured by qRT-PCR and Western blotting. The results showed that 500 μg/ml APS significantly inhibited the LPS-induced increase of IL-6 mRNA and protein in THP-1 macrophages (Figures 1A–C and Supplementary Figure S2). We further examined the effects of APS treatment time (12, 24 and 48 h) on LPS-induced IL-6 expression in THP-1 macrophages. The results showed that APS treatment for 24 h had the most obvious inhibitory effect on IL-6 (Figures 1D–F). These results indicated that the inhibitory effect of APS on IL-6 expression in THP-1 macrophages was concentration- and time-dependent.

### APS abrogated LPS-induced m^6^A methylation in THP-1 macrophages

**FIGURE 2:**
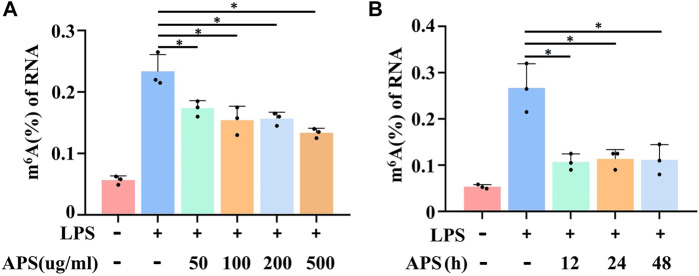
APS abrogated LPS-induced m^6^A level in THP-1 macrophages. **(A)** THP-1 macrophages were treated with APS (50, 100, 200, 500 μg/ml) for 24 h and then induced with LPS (10 μg/ml) for 4 h m^6^A level was detected in the cells by m^6^A RNA methylation quantification. **(B)** THP-1 macrophages were treated with APS (500 μg/ml) for different time (0, 12, 24, 48 h) and then induced with LPS (10 μg/ml) for 4 h. m^6^A level was detected in the cells by m^6^A RNA methylation quantification. ^*^ *p* < 0.05 compared with LPS group. Data represent the mean ± SEM. n = 3 per group.

Recent evidence suggests that m^6^A methylation plays a crucial role in regulating THP-1 macrophage inflammation (Wang et al., 2019), but whether APS plays a role in the m^6^A modification is unclear. To investigate the effect of APS on m^6^A methylation, m^6^A levels in APS-treated THP-1 macrophages were measured by an m^6^A quantification kit. The results showed that LPS-induced m^6^A methylation was decreased with increasing APS concentrations (Figure 2A). Furthermore, we examined the m^6^A modification level after stimulation with APS for 12, 24 and 48 h, and the results indicated that the level of m^6^A was significantly lower than that in the LPS-treated group (Figure 2B). These results suggest that APS regulates m^6^A modification levels in THP-1 macrophages. Then, we treated LPS-induced THP-1 macrophages with 500 μg/ml APS for 24 h for the following mechanistic studies.

### APS decreased LPS-induced WTAP gene expression in THP-1 macrophages

**FIGURE 3:**
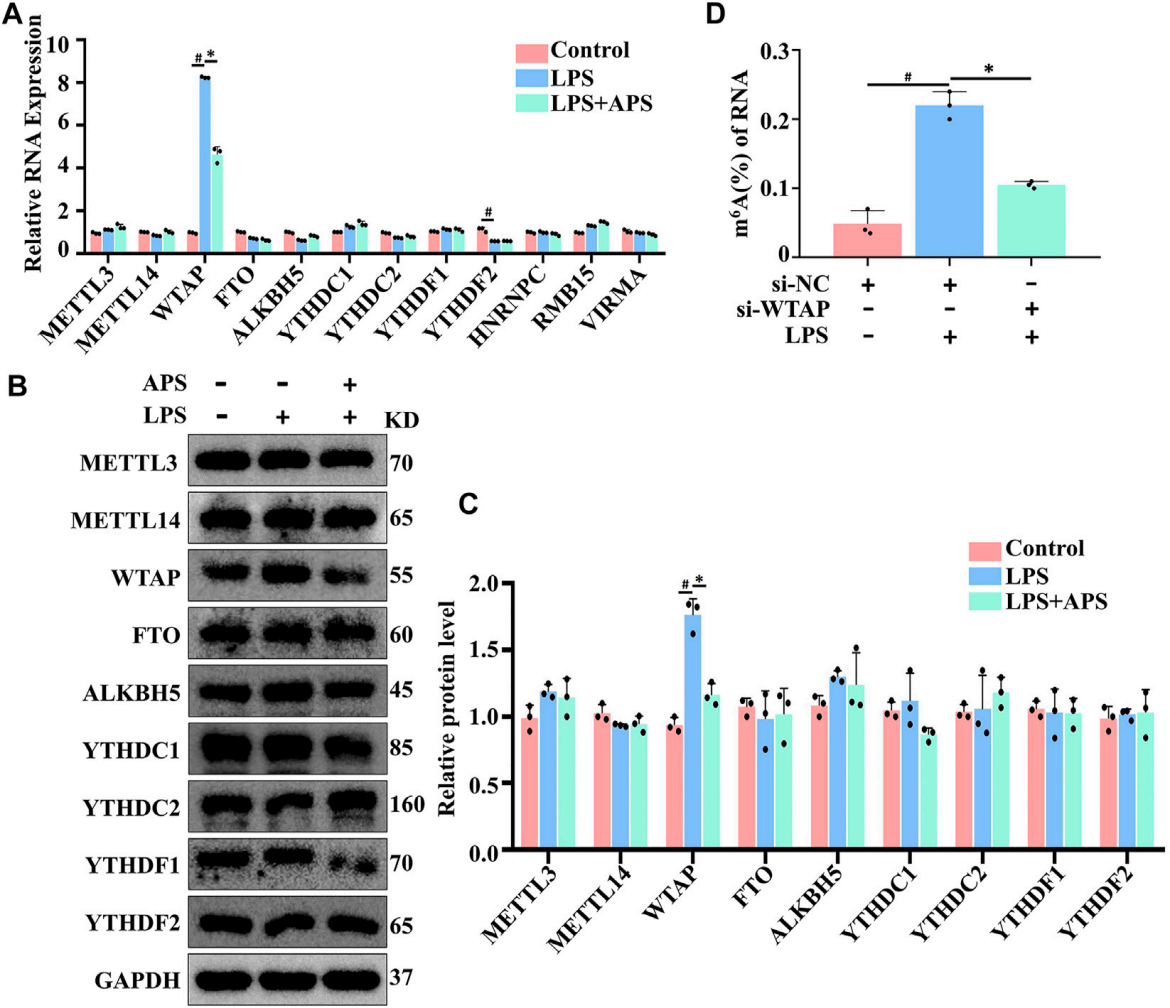
APS decreased LPS-induced WTAP gene in THP-1 macrophages. THP-1 macrophages were treated with APS (500 μg/ml) for 24 h and then induced with LPS (10 μg/ml) for 4 h. m^6^A modification system-related proteins were detected by **(A)** qRT-PCR and **(B)** western blot. **(C)** Densitometry quantification of protein. **(D)** THP-1 macrophages were transfected with si-WTAP and then induced with LPS (10 μg/ml) for 4 h. Overall m^6^A level in RNA is detected by m^6^A RNA methylation quantification. ^#^ *p* < 0.05 compared with Control group. ^*^ *p* < 0.05 compared with LPS group. Data represent the mean ± SEM. n = 3 per group.

To examine the mechanism by which APS regulates m^6^A modification levels, we examined whether APS treatment altered m^6^A modification system-related protein expression in LPS-induced THP-1 macrophages. As shown in Figure 3A, WTAP mRNA expression was markedly decreased after treatment with APS for 24 h. The protein level of WTAP was also significantly decreased compared with that of the other related proteins (Figures 3B,C). WTAP is one of the essential proteins in the m^6^A methyltransferase complex and plays an important role in m^6^A writing. To verified the relationship between WTAP and m^6^A levels. We knocked down WTAP in LPS-treated THP-1 macrophages with siRNA. The results showed that WTAP knockdown suppressed the LPS-induced increase in total m^6^A levels in THP-1 macrophages. Taken together, these data suggest that APS can affect m^6^A modification levels by regulating WTAP expression (Figure 3D).

### APS exerted an anti-inflammatory effect by regulating WTAP expression

**FIGURE 4:**
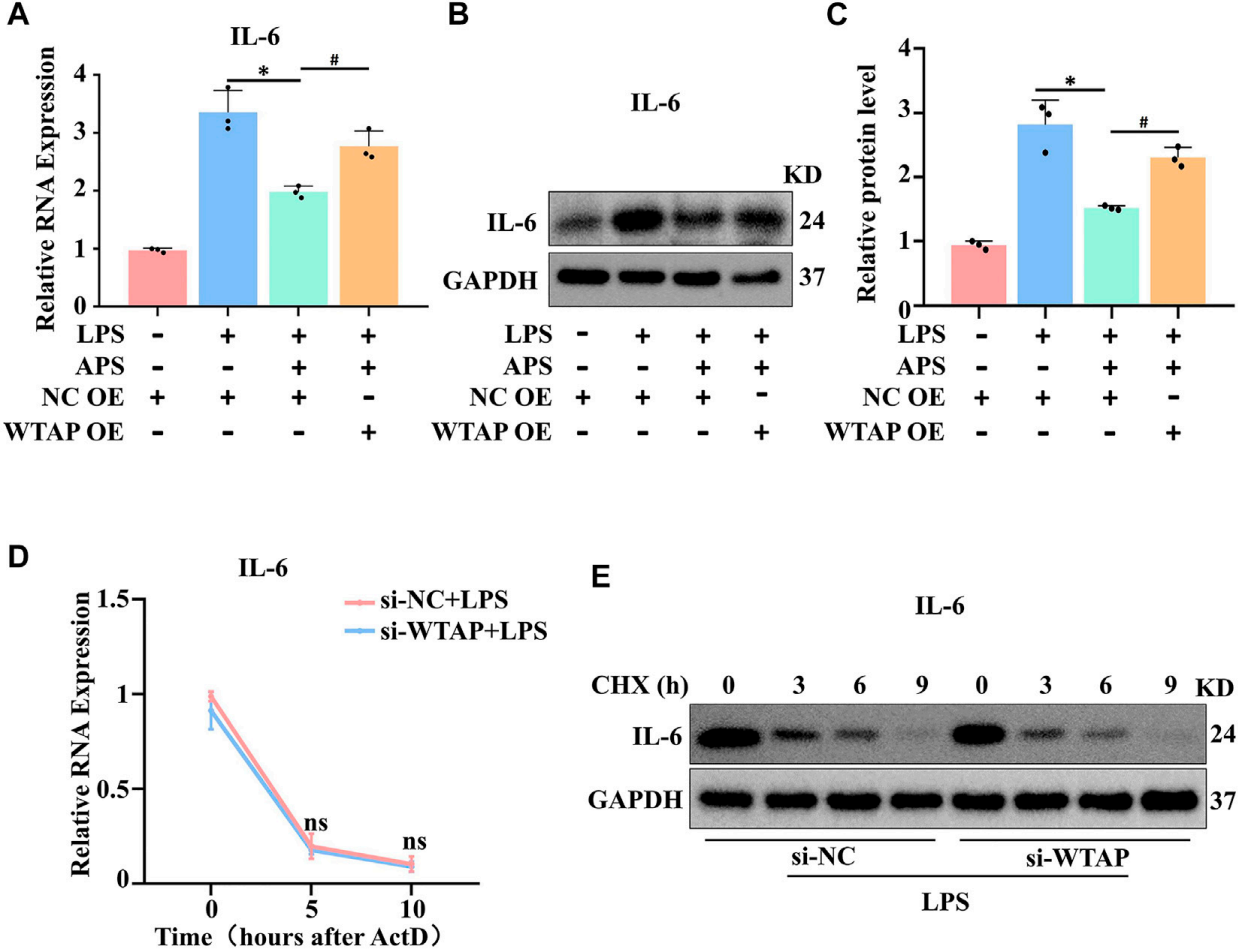
APS exerted an anti-inflammatory effect by regulating WTAP expression. IL-6 expression in APS-treated THP-1 macrophages after WTAP overexpression was detected by **(A)** qRT-PCR and **(B)** western blot. **(C)** Densitometry quantification of protein. **(D)** IL-6 mRNA stability following treatment with actinomycin D (ActD, 2 μM) was detected by qRT-PCR. **(E)** THP-1 macrophages were treated with cycloheximide (CHX, 10 μM) after si-WTAP transfection. IL-6 protein was detected by western blot. ^*^ *p* < 0.05 compared with NC OE + LPS group. ^#^ *p* < 0.05 compared with NC OE + LPS + APS group. Data represent the mean ± SEM. n = 3 per group.

To verify that APS affects the expression of IL-6 through WTAP, we examined the mRNA and protein expression of IL-6 by qRT‒PCR and western blotting, respectively, after WTAP overexpression. WTAP overexpression ameliorated the low expression of IL-6 in APS-treated THP-1 macrophages (Figures 4A–C). Next, we identified the mechanism by which WTAP regulates IL-6. Because WTAP is one of the essential proteins in the m^6^A writer, we hypothesized that WTAP-mediated regulation of IL-6 expression may be dependent on the m^6^A modification. Interestingly, we predicted the m^6^A modification sites of IL-6 with SRAMP website (http://www.cuilab.cn/sramp) and found that there were multiple m^6^A modification sites (GGAC) in the IL-6 mRNA sequence (Supplementary Table S3), suggesting that IL-6 mRNA can undergo m^6^A modification. The main mechanism by which m^6^A modification regulates protein expression is by regulating mRNA stability, translation shearing and nuclear transport. Among these, m^6^A modification most commonly regulates the stability of mRNA and protein. Therefore, we first examined the stability of IL-6 mRNA. As shown in Figure 4D, LPS stimulation and WTAP knockdown had no effect on the stability of IL-6 mRNA. We further examined protein levels after treatment with CHX to stop translation. The results revealed that knockdown of WTAP had no effect on the stability of IL-6 protein in THP-1 macrophages (Figure 4E). Therefore, these results indicated that WTAP does not regulate IL-6 through m^6^A modifications.

### APS regulated IL-6 expression through WTAP-mediated p65 nuclear translocation

**FIGURE 5:**
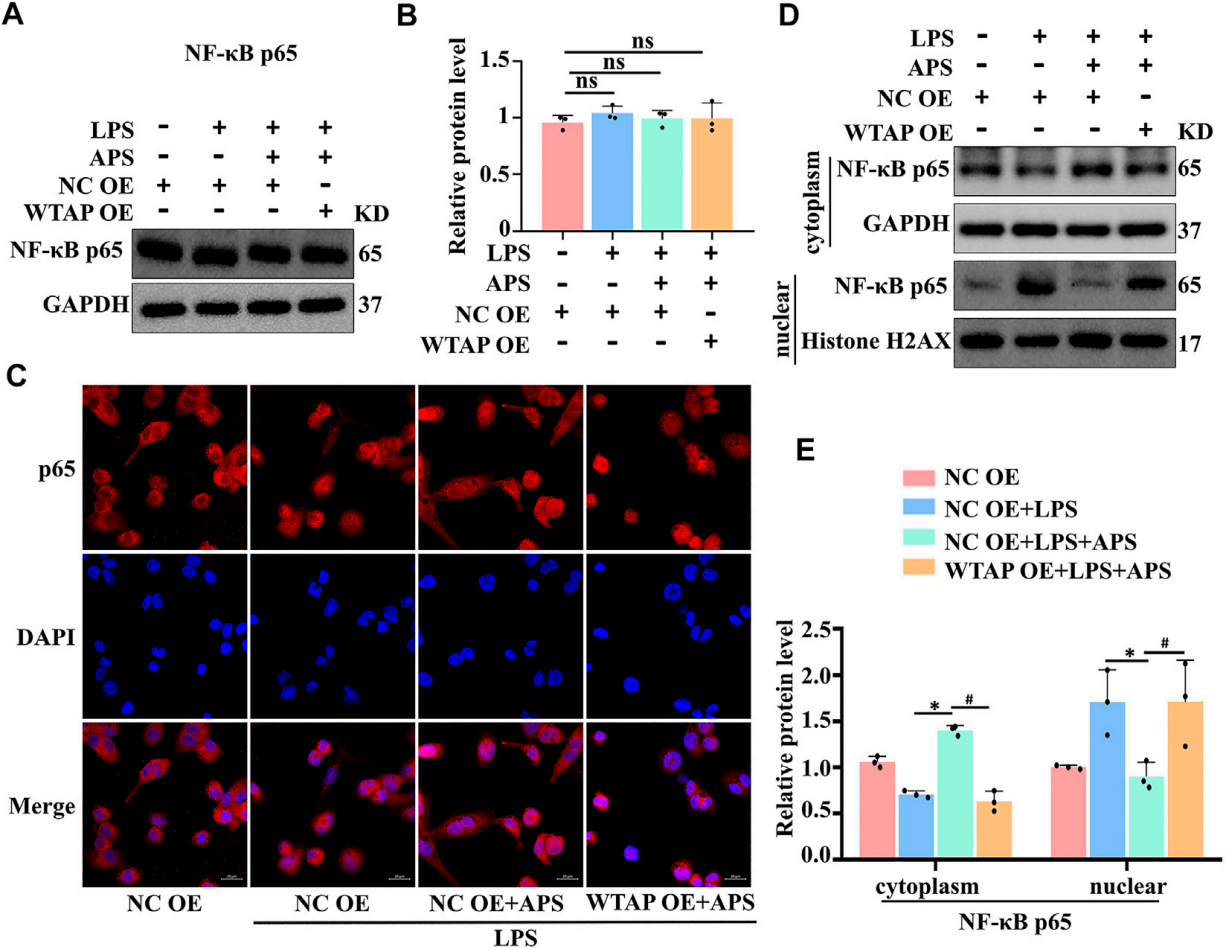
APS regulates IL-6 expression through WTAP-mediated p65 nuclear translocation. THP-1 macrophages were transfected with WTAP overexpression plasmid prior to treatment with APS. **(A)** NF-κb p65 protein expression was detected by western blot. **(B)** Densitometry quantification of protein. **(C)** The localization of NF-κb p65 (red) was observed with immunofuorescence, and the nuclei (blue) were stained with DAPI, scale bar, 20 μm. **(D)** The nuclear translocation of p65 was analyzed, and its localization in the cytosol and nucleus was separately quantified by western blot. **(E)** Densitometry quantification of protein. ^*^ *p* < 0.05 compared with NC OE + LPS group. ^#^ *p* < 0.05 compared with NC OE + LPS + APS group. Data represent the mean ± SEM. n = 3 per group.

Therefore, how does WTAP regulate the expression of IL-6? Multiple studies have shown that NF-κB p65 can regulate transcription factors associated with IL-6 expression (Wang et al., 2019; Jiang et al., 2022). We hypothesized that WTAP regulates IL-6 through the NF-κB pathway and examined the expression level of p65 in APS-treated THP-1 macrophages by western blotting. The results showed that APS did not affect the expression of p65 (Figures 5A,B). WTAP overexpression did not affect the overall expression level of p65 in THP-1 macrophages (Figures 5A,B, Supplementary Figure S3). We further examined the nuclear transfer of p65. Immunofluorescence staining showed that APS prevented LPS-induced translocation of p65 from the cytoplasm to the nucleus in THP-1 macrophages (Figure 5C). However, WTAP overexpression promoted the translocation of p65 from the cytoplasm to the nucleus in APS-treated THP-1 macrophages (Figure 5C). To better examine the nuclear and cytoplasmic distribution of p65, we extracted nuclear and cytoplasmic proteins from THP-1 macrophages. The results showed that APS-mediated inhibition of p65 translocation to the nucleus was reversed in THP-1 macrophages that were transfected with the WTAP overexpression plasmid (Figures 5D,E). These results suggest that APS regulates IL-6 expression through WTAP-mediated p65 nuclear translocation.

## Discussion

APS has attracted the attention of researchers because of its anti-inflammatory, antioxidant and immunomodulatory effects. Studies have shown that APS exerts its anti-inflammatory effect by inhibiting LPS-induced production of TNF-α and IL-8 in intestinal epithelial cells by blocking mitogen-activated protein kinase (MAPK) transcriptional activity (Yuan et al., 2009). In addition to identifying the role of APS through its anti-inflammatory and antioxidant properties, recent studies have demonstrated a link between APS and the DNA methylome (Liu et al., 2021). In this study, we verified that APS exerts its anti-inflammatory effect on THP-1 macrophages through m^6^A modifications.

The m^6^A is the most widely studied RNA modification and have been recognized as a new layer of epigenetic regulation, which is closely related to the occurrence and development of macrophage inflammation. Recent studies have shown that the level of the m^6^A modification is significantly increased in LPS-stimulated THP-1 macrophages, and knocking down the key m^6^A modification enzyme METTL14 inhibits the occurrence of macrophage inflammation (Zheng et al., 2022). In our study, we also found that the m^6^A modification level was significantly increased in LPS-stimulated THP-1 macrophages, and the m^6^A modification level decreased with the concentration increasing of APS. These results suggest that APS can regulate the m^6^A modification.

In recent clinical trials, ziltivekimab, a novel IL-6 ligand inhibitor, was highly effective in reducing inflammatory responses and biomarkers of atherosclerosis, suggesting that IL-6 is an effective therapeutic target for atherosclerosis (Ridker and Rane, 2021). Our findings suggested that IL-6 is a target of WTAP in APS-treated THP-1 macrophages. Since WTAP is one of the essential proteins for m^6^A writing, we hypothesize that WTAP-mediated regulation of IL-6 expression may depend on m^6^A modifications. However, our results demonstrated that the stability of IL-6 mRNA and protein were not significantly changed in WTAP knockdown cells. The m^6^A modifications on mRNA are mainly written by the METTL3, METTL14 and WTAP complexes (Xu et al., 2022), and the evidence shown that METTL14 does not regulate IL-6 through m6A modification (Zheng et al., 2022). This evidence shows that WTAP does not directly regulate IL-6 through m^6^A modification.

Multiple studies have shown that NF-κB p65 can regulate the transcription of IL-6. As expected, WTAP overexpression reversed the distribution of p65 in the cytoplasm in APS-treated THP-1 macrophages, which regulated the transcription of IL-6. Moreover, we examined the expression of other m^6^A enzymes upon overexpression of WTAP, and the results suggested that WTAP could not induce complementary effects of other m^6^A writers (Supplementary Figure S4).In conclusion, the therapeutic mechanism of APS involves regulating IL-6 expression through WTAP-mediated p65 nuclear translocation.

This study has certain limitations. First, the underlying mechanism by which WTAP regulates the nucleocytoplasmic distribution of p65 needs to be further examined. Second, our entire study was performed at the cellular level, and further validation of the results in mice is needed.

In conclusion, our findings confirmed that APS could exert an anti-inflammatory effect in THP-1 macrophages. We demonstrated that APS could reduce the m^6^A modification level. To the best of our knowledge, this is the first study on the m^6^A modification in the anti-inflammatory mechanism of APS. Mechanistically, we showed that WTAP regulated the expression of IL-6 by regulating p65 nuclear translocation. Our findings reveal a novel role for APS in the regulation of the m^6^A modification during macrophage inflammation, and this has great potential for macrophage inflammation therapy.

## Data Availability

The datasets presented in this article are not readily available because N.A. Requests to access the datasets should be directed to zhaoguojun@gzhmu.edu.cn.
