# Supplementary material for: WTAP mediates the anti-inflammatory effect of Astragalus mongholicus polysaccharide on THP-1 macrophages
Source: Front Pharmacol. 2022 Oct 7;13:1023878. doi: 10.3389/fphar.2022.1023878 (PMC9585178; doi:10.3389/fphar.2022.1023878)
Supplement: Supplementary file 1 [file DataSheet2.docx]

Raw Data (Figures and Supplementary Figures): https://www.jianguoyun.com/p/DY43kQ0Q3t35Chit_dgEIAA
