# Supplementary material for: WTAP mediates the anti-inflammatory effect of Astragalus mongholicus polysaccharide on THP-1 macrophages
Source: Front Pharmacol. 2022 Oct 7;13:1023878. doi: 10.3389/fphar.2022.1023878 (PMC9585178; doi:10.3389/fphar.2022.1023878)
Supplement: Supplementary file 2 [file DataSheet1.docx]

**SUPPLEMENTARY MATERIAL**

**Title：WTAP mediates the anti-inflammatory effect of astragalus mongholicus polysaccharide in THP-1 macrophages**

**Table 1. Sequences of qRT-PCR primers.**

| **Gene** | **Species** | **Forward primer (5’-3’)** | **Reverse primer (5’-3’)** |
| --- | --- | --- | --- |
| METTL3 | Human | ACCTCAGTGGATCTGTTGTG | CTCCATGGCCCTGCCTGTGA |
| METTL14 | Human | GGACCTTGGAAGAGTGTGTTT | GGCAGTGTTCCTTTGTTCTC |
| WTAP | Human | GAGTGTACTACTCAAATCCA | CCCTGTTTGGCTATCAGGCG |
| YTHDC1 | Human | AGAGTCATATGCAGGTTCAGA | GGACCATACACCCTTCGCTT |
| YTHDC2 | Human | TATAAAAAGATTCCTCCAG | ATGCCATCCACTCTAAAGGAT |
| YTHDF1 | Human | TGTTCATGAAGCATGTCGGCC | TAACTGTTACTCTGATTTGA |
| YTHDF2 | Human | TTCCGCGTGGACGGGGCTCA | CCTTTTGGTCTCTGCTCCAAG |
| FTO | Human | TAGCTGTGAAGGCCCTGAAGA | AGATCATCAAGCATGAAATAG |
| ALKBH5 | Human | ATCCTCAGGAAGACAAGATT | ATCCGTGGCCTGTGGGCAGC |
| HNRNPC | Human | TATTATGATAGGATGTACAGT | TCCTTTCAACTTTCCAGACT |
| RBM15 | Human | GCAATTGGCCAATGAGGACT | GAGTCCTCACCACCACGGGA |
| IL-6 | Human | TTTTCCCACCATCTTTCCTC | CTGGCTTGTTCCTCACTACTCT |

**Table 2 Antibody for Immunofluorescence/western Blotting.**

| **Antibody Target** | **Dilution** | **Clone^#^** | **Supplier** |
| --- | --- | --- | --- |
| IL-6 | 1:1000 | #DF6087 | Affinity |
| METTL3 | 1:1000 | Cat#86132 | Cell Signaling |
| METTL14 | 1:1000 | Cat#51104 | Cell Signaling |
| WTAP | 1:1000 | E705B | Cell Signaling |
| YTHDF1 | 1:1000 | Cat#86436 | Cell Signaling |
| YTHDF2 | 1:1000 | Cat#80014 | Cell Signaling |
| YTHDC1 | 1:1000 | Cat#ab220159 | Abcam |
| YTHDC2 | 1:1000 | Cat#ab220160 | Abcam |
| FTO | 1:1000 | Cat#31687 | Cell Signaling |
| ALKBH5 | 1:1000 | Cat#ab195377 | Abcam |
| hnRNP C | 1:1000 | Cat#91327 | Cell Signaling |
| NF-κB p65 | 1:1000 | ab16502 | abcam |
| GAPDH | 1:1000 | #AF7021 | Affinity |
| Histone H2AX | 1:1000 | M020534S | Abmart |
| Anti-rabbit secondary antibody | 1:30000 | BS13278 | Bioworld |
| Anti-mouse secondary antibody | 1:2000 | ab6789 | abcam |

**Table 3. Prediction of N6-methyladenosine (m^6^A) modification sites based on the IL-6 sequence.**

| **#** | **Position** | **Sequence context** | **Structural context** | **Local structure visualization** | **Score(binary)** | **Score(knn)** | **Score(spectrum)** | **Score(combined)** | **Decision** |
| --- | --- | --- | --- | --- | --- | --- | --- | --- | --- |
| 1 | 579 | UGAGG CCAAC GGGGC CGACU AGACU GACUU CUGUA UUUAU CCUUU | N/A | N/A | 0.631 | 0.427 | 0.472 | 0.557 | m^6^A site (**Low** confidence) |
| 2 | 767 | UUGUC CCCCG GGCUU UGGAA GGACU AGAAA AGUGC CACCU GAAAG | N/A | N/A | 0.726 | 0.777 | 0.483 | 0.631 | m^6^A site (**High** confidence) |
| 3 | 1095 | CCAUC AGUAA AAUUG GGCGU GGACU AGGUG AUCUC AUAGA UCCUU | N/A | N/A | 0.665 | 0.663 | 0.507 | 0.601 | m^6^A site (**Moderate** confidence) |
| 4 | 1276 | UUUUG GAGAU AAGGA AACUG AGACU CAGGA UUAAG UAACA CACCU | N/A | N/A | 0.636 | 0.581 | 0.569 | 0.606 | m^6^A site (**Moderate** confidence) |
| 5 | 1320 | UAAAG UCACA GGUGA GCUUG GAACU GAACC CAAGU GUGCC CCCAC | N/A | N/A | 0.671 | 0.643 | 0.642 | 0.658 | m^6^A site (**High** confidence) |
| 6 | 1400 | UGUAG CUUCA UUUUU CUUAG AGACU UUCCU GGCUG UGGUU GAACA | N/A | N/A | 0.694 | 0.48 | 0.411 | 0.570 | m^6^A site (**Low** confidence) |
| 7 | 2239 | GAGCA GAGGG AAAAG AUGUC GAACU GUGGC AAUUU UAACU UUUCA | N/A | N/A | 0.678 | 0.542 | 0.528 | 0.611 | m^6^A site (**Moderate** confidence) |
| 8 | 2514 | GUGGG GGAAG ACAGG CUCAA AGACA GUGUC CUGGA CAACU CAGGG | N/A | N/A | 0.607 | 0.554 | 0.565 | 0.587 | m^6^A site (**Moderate** confidence) |
| 9 | 2526 | AGGCU CAAAG ACAGU GUCCU GGACA ACUCA GGGAU GCAAU GCCAC | N/A | N/A | 0.580 | 0.653 | 0.608 | 0.595 | m^6^A site (**Moderate** confidence) |
| 10 | 2929 | UAGUU CAGAG AAAUG GUCAG AGACU CAAGG GUGGA AAGAG GUACC | N/A | N/A | 0.708 | 0.730 | 0.481 | 0.618 | m^6^A site (**Moderate** confidence) |
| 11 | 3468 | UUCAU UCAUA UACUA AAUAU GAACU AUGUG CCAGG CAUUA UUUCA | N/A | N/A | 0.690 | 0.693 | 0.433 | 0.587 | m^6^Asite (**Moderate** confidence) |
| 12 | 4448 | AAACC UGUCC ACUGG GCACA GAACU UAUGU UGUUC UCUAU GGAGA | N/A | N/A | 0.672 | 0.497 | 0.581 | 0.627 | m^6^A site (**High** confidence) |

**Supplementary Legends**

**Supplementary Figure 1. MTT assay shows cell viability in 50-500 μg/mL APS-treated THP-1 macrophages. Data represent the mean ± SEM. n=3 per group.**

**Supplementary Figure 2. The alterations of inflammatory cytokines in APS-treated LPS-induced THP-1 macrophages.**

THP-1 macrophages were treated with APS (50, 100, 200, 500 μg/mL) for 24 h and then induced with LPS (10μg/ mL) for 4 h. The mRNA of IL-18 (A), IL-1β (B), TNF-α (C) and NLRP3 (D) was detected by qRT-PCR. (E) IL-18, IL-1β, TNF-α and NLRP3 protein was detected by western blot. ^*^*P* < 0.05 compared with LPS group. Data represent the mean ± SEM. n=3 per group.

**Supplementary Figure 3. The silencing and overexpression efficiency of WTAP.**

The transfection efficiency of siRNA was detected by qRT-PCR (A) and western blot (B). (C) Densitometry quantification of protein. ^*^*P* < 0.05 compared with si-NC group. The transfection efficiency of overexpression plasmid was detected by qRT-PCR (D) and western blot (E). (F) Densitometry quantification of protein. ^*^*P* < 0.05 compared with NC OE group. Data represent the mean ± SEM. n=3 per group.

**Supplementary Figure 4. The effects of overexpressing WTAP on other m6A enzymes in THP-1 macrophages.** m^6^A enzymes expression in APS-treated THP-1 macrophages after WTAP overexpression was detected by qRT-PCR (A) and western blot (B). Data represent the mean ± SEM. n=3 per group.

.
